# Drainage by Ovarian Incision for the Treatment of Massive Ovarian Edema Torsion During Pregnancy

**DOI:** 10.7759/cureus.60194

**Published:** 2024-05-13

**Authors:** Haruhiko Shimizu, Kiguna Sei, Aimi Oda, Yumi Shimizu, Hiroshi Adachi

**Affiliations:** 1 Obstetrics and Gynecology, Seirei Hamamatsu General Hospital, Hamamatsu, JPN

**Keywords:** ovulation induction, laparoscopy, massive ovarian edema, ovarian torsion, clomiphene citrate, pregnancy

## Abstract

Massive ovarian edema (MOE) is a rare benign condition presenting as unilateral ovarian enlargement with stromal edema, and only a limited number of MOE cases during pregnancy have been reported. MOE is often complicated by ovarian torsion, which requires detorsion. Although the diagnosis of MOE can be made using ultrasound and magnetic resonance imaging, its rarity makes diagnosis difficult, usually leading to overtreatment. Preserving the ovary in the treatment of MOE torsion is essential, and consideration of oophoropexy after detorsion is often reported. However, fixing an enlarged ovary to the pelvic wall in the limited space of the pelvis is challenging.

Herein, we present a case of MOE of the right ovary diagnosed at the fifth week of gestation after ovulation induced by clomiphene citrate. Torsion of the ovary occurred in the seventh week. We achieved preservation of the ovary through laparoscopic surgery with detorsion and drainage by making a small incision to the enlarged ovary, resulting in an immediate size reduction. There was no recurrence of torsion or MOE throughout the pregnancy, and the patient gave birth in the 39th week of gestation. This is the third reported case of MOE after ovulation using clomiphene citrate, and it highlights the effectiveness of treatment with detorsion and a small incision of the ovary via laparoscopic surgery in patients with MOE torsion during pregnancy.

## Introduction

Massive ovarian edema (MOE) is an uncommon but benign condition characterized by unilateral ovarian enlargement with stromal edema and usually occurs in young women. Approximately 200 cases of MOE have been reported previously. Moreover, MOE during pregnancy is rare, with, to the best of our knowledge, only 13 cases reported to date. The etiology is unknown but is speculated to involve venous and lymphatic drainage obstruction caused by partial or complete ovarian torsion [1]. Enlargement of the ovary may contribute to the likelihood of partial torsion. There have been two reports of MOE caused by ovulation induced by clomiphene citrate [2,3]. It is crucial to differentiate MOE from other ovarian tumors due to its benign nature, which makes ovary preservation, an essential aim in young or pregnant women, possible. Although the diagnosis of MOE can be made using ultrasound and magnetic resonance imaging (MRI) with its characteristic findings, its rarity makes diagnosis difficult, often leading to overtreatment such as oophorectomy [4,5]. For the treatment of MOE torsion, detorsion of the ovary is essential to relieve pain, and drainage of the edema is expected to occur in time, since the obstruction is released anatomically. Performing oophoropexy after detorsion is one way of preventing recurrence. However, fixing an enlarged ovary to the pelvic wall in the limited space of the pelvis is challenging, and an alternative method is to perform a wedge resection to drain the edema [5].

We report the third case of MOE occurring after the use of clomiphene citrate accompanied by ovarian torsion. The patient was treated successfully with laparoscopic surgery with the aim of preserving the ovary. Accurate preoperative diagnosis was facilitated through the utilization of ultrasound and MRI.

## Case presentation

A woman in her late 20s with a history of irregular menstrual periods due to polycystic ovarian syndrome (PCOS) underwent reproductive treatment at a clinic. For ovarian stimulation, 50 mg of clomiphene citrate was administered for five days starting from the fifth day of the menstrual cycle. On day 16 of the cycle, ovulation was induced with human chorionic gonadotropin, and timed intercourse was facilitated. Double ovulation from the right ovary was confirmed by the disappearance of two follicles. At this time, no apparent sign of ovarian enlargement was noted.

The patient returned to the clinic after surpassing the expected date of her menstrual cycle. She was at approximately five weeks of gestation. A gestational sac (GS) was observed in the uterus. For the first time, enlargement of the right ovary with a large hematoma-like, ring-shaped structure was detected. Due to the suspicion of heterotopic pregnancy, the patient was referred to our hospital for further examination.

**Figure 1:**
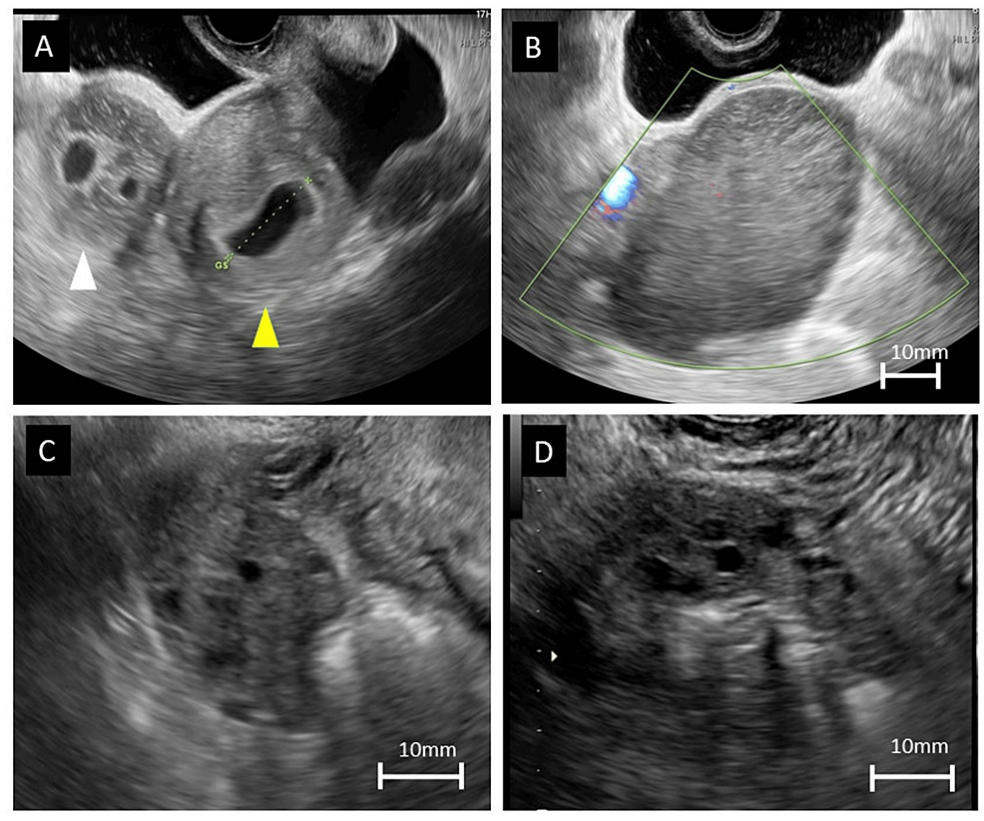
Transvaginal ultrasound imaging and the time course of ovarian changes A: Image of the preoperative ovary. Enlargement of the right ovary can be observed (white arrowhead), whereas the gestational sac is detected in the uterine cavity (yellow arrowhead). The right ovary measures 93x70 mm. B: Image of the preoperative ovary. Color Doppler imaging of the right ovary shows no abnormal blood flow. C: Image of the ovary at the one-week follow-up after discharge. The right ovary measures 37x24 mm. D: Image of the ovary at one month after delivery. The right ovary measures 23x17 mm.

During the initial examination, the patient did not report abdominal distension or tenderness. Transvaginal ultrasonography revealed a uterus measuring 82 mm, with the GS identified in the uterine cavity. However, no fetal heartbeat was detected at that time. The right ovary was enlarged, measuring 70×93 mm. The internal structure exhibited high echogenicity with relatively uniform findings, and multiple follicles were observed at the periphery of the ovary. No irregularities resembling a hematoma were noted in the internal echo, and neither GS nor abnormal blood flow was detected (Figure 1A).

A small amount of ascitic fluid was also observed. Considering the clinical course and relatively low likelihood of a heterotopic pregnancy, the possibility was deemed to be low. Furthermore, regarding the findings, the possibility of a rapidly growing solid tumor or non-tumor condition, such as MOE, was suspected. Because the patient was asymptomatic, a cautious outpatient observation approach was chosen.

Two days after the initial visit, the patient returned for an outpatient follow-up. She had no specific symptoms, and the ovary size remained the same. The tumor marker levels were as follows: carcinoembryonic antigen (CEA), undetectable; cancer antigen (CA) 125, 228.9 U/mL; CA19-9, 3.81 U/mL; and alpha-fetoprotein, 3.75 ng/mL. On a follow-up visit 10 days later, ultrasonography indicated a slight reduction in the size of the right ovary to 60×70 mm. A GS equivalent to seven weeks and zero days of gestation, along with the fetal heartbeat, was observed in the uterus.

The following morning, the patient experienced a sudden onset of lower right abdominal pain. Tenderness was observed over the enlarged right ovary. Ultrasonography revealed enlargement of the right ovary to 80x60 mm (Figure 1B). A slight increase in fluid accumulation was observed in the Douglas pouch, and spiral-shaped ovarian vessels were detected using color Doppler imaging. The patient's pain improved after administration of 1000 mg of acetaminophen. In the absence of elevated inflammatory markers in blood tests, ovarian torsion was diagnosed, and the patient was admitted for management. Considering the substantial impact of an ovarian enlargement diagnosis on the treatment strategy and pregnancy outcomes, confirming the diagnosis using additional MRI was deemed necessary. Although MRI safety in early pregnancy has not yet been firmly established, we thoroughly explained to the patient that the benefits of the examination outweighed the potential risks. Written informed consent was obtained from the patient before proceeding with this study.

**Figure 2:**
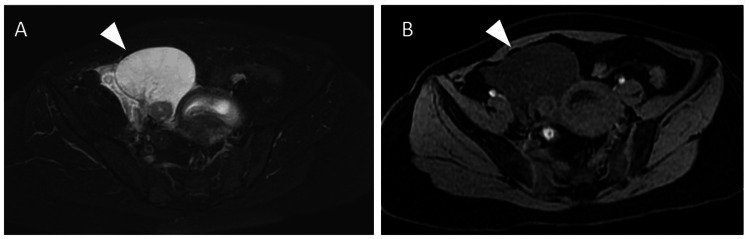
Pelvic magnetic resonance imaging findings A: Axial T2-weighted image. A high signal is observed in the stromal region of the right ovary. B: Axial T1-weighted image. No sign of bleeding or hematoma in the right ovary is evident.

On T2-weighted images, a high signal was observed in the stromal region, and a teardrop configuration was identified. T1-weighted imaging did not reveal any high-intensity findings or evidence of bleeding (Figure 2).

Based on medical history, ultrasound findings, and MRI results, a diagnosis of torsion of the right ovary due to MOE was confirmed. Considering that ovulation occurred in the right ovary and the patient strongly desired to continue the pregnancy, a conservative surgical approach was adopted. In addition, to prepare for any unforeseen circumstances, biopsy by small incision was planned for pathological diagnosis according to the guidelines for Gynecological Practice in Japan [6]. A second surgery was considered in case malignant findings or other concerns were noted.

**Figure 3:**
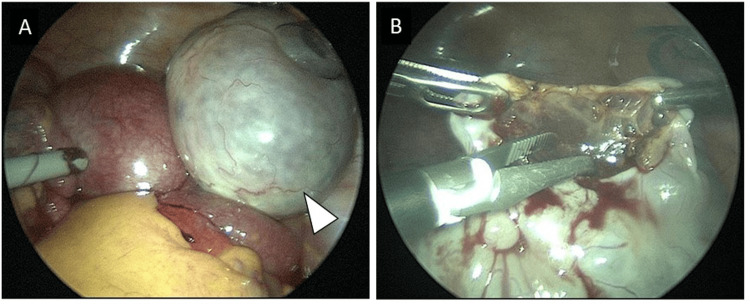
Laparoscopic findings A: Enlargement of the right ovary is observed with a smooth, white, and edematous surface undergoing 360° clockwise torsion (white arrowhead). B: Small incision for biopsy of the right ovary placed opposite from the ovarian vessels, with observable edematous stroma.

The surgery was minimally invasive, with a low pneumoperitoneum pressure of 8 mmHg for laparoscopic surgery. A 5-mm port was placed at the umbilicus to observe the abdominal cavity. Upon exploration, the right ovary was found to occupy the pelvic cavity, undergoing a 360° clockwise torsion. The surface of the right ovary appeared smooth, white, and edematous (Figure 3A).

No abnormalities were observed in the left ovary. Three additional ports were added to the lower abdominal quadrant, and the torsion was successfully corrected. After torsion correction, biopsies were performed via small incisions using a monopolar instrument at two sites for diagnostic purposes (Figure 3B). Leakage of serous fluid from the incision occurred during traction of the ovary, and the size of the ovary nearly equalized with the left side within 10 minutes during hemostatic maneuvers. Postoperatively, the fetal heartbeat was confirmed, and no sign of impending complications was noted.

**Figure 4:**
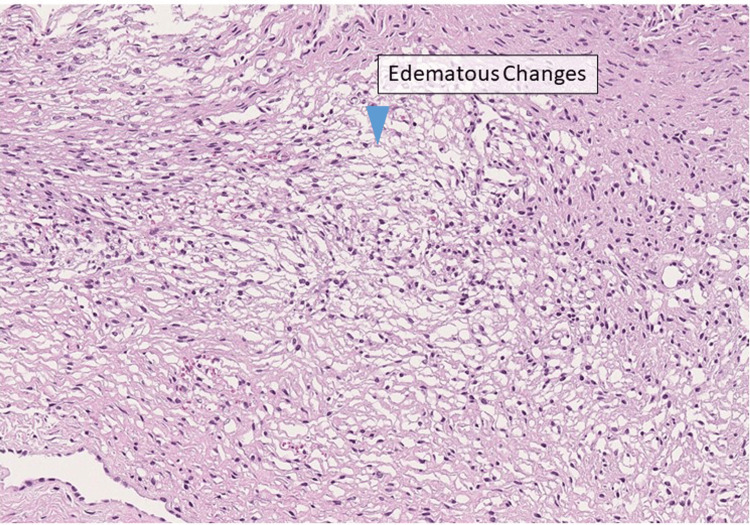
Histopathological findings An increase of spindle-shaped stromal-like cells in the ovary is observed, accompanied by edematous changes in the stroma (blue arrowhead). No malignant findings are noted (low-power field; hematoxylin-eosin stain).

The histopathological findings indicated edematous changes in the stroma without evidence of malignancy (Figure 4), confirming the diagnosis of MOE torsion.

One day postoperatively, the right ovary reduced in size to 47x32 mm. The patient's postoperative recovery was uneventful, and she was discharged three days postoperatively. At the one-week follow-up after discharge, no recurrence of ovarian enlargement was observed (Figure 1C). Throughout the first and early second trimesters of pregnancy, efforts were made to monitor the ovarian condition as closely as possible, and no sign of recurrence was noted.

The subsequent course of the pregnancy was uneventful, with no fetal anomalies or complications. At 39 weeks and two days of gestation, the patient experienced spontaneous rupture of the membranes, followed by natural labor. Due to prolonged labor, forceps delivery was performed. Postpartum examination revealed no abnormalities in the newborn. Ovarian follow-up via ultrasound was conducted at discharge, as well as at one and three months postpartum, confirming no recurrence of ovarian enlargement (Figure 1D), and follow-up was concluded.

## Discussion

We experienced a case of MOE after administration of clomiphene, which resulted in ovarian torsion and required surgical treatment in the first trimester of pregnancy. This is the third case of MOE after administration of clomiphene and the second case of MOE during pregnancy that occurred in a patient who had received clomiphene citrate for ovulation induction.

MOE is a rare but benign condition characterized by unilateral ovarian enlargement with stromal edema. MOE occurs due to venous and lymphatic reflux abnormalities caused by partial ovarian torsion [7,8]. During pregnancy, reported cases are diagnosed between 11 and 32 weeks of gestation [4], and chronic vascular congestion due to gravid uterus is assumed to be responsible for some cases [9]. Its risk has also been reported to increase when lesions are present in the ovary. Similar to the present case, there have been several prior reports of MOE development in patients treated for PCOS with clomiphene [2,3]. In this case, we concluded that ovarian enlargement due to ovarian stimulation with clomiphene citrate in the presence of PCOS led to partial torsion of the ovarian corpus luteum cyst, ultimately resulting in MOE.

MOE can be complicated by torsion, as reported in 43-59% of surgically treated cases [3,5]. In MOE during pregnancy, the frequency of torsion is approximately the same [4]. In contrast, reports have suggested that even during pregnancy, a conservative approach can lead to improvement, provided no symptoms are present [10]. Accordingly, considering the ongoing pregnancy at the time of diagnosis, we initially selected a cautious observational approach.

Differential diagnosis is especially essential in the treatment of ovarian enlargement during pregnancy, since removal of the ovary would affect future fertility. Characteristic ultrasound findings of MOE include a solid tumor-like mass and multiple ovarian follicles located around the periphery of the cortex of the enlarged ovary [7,11]. Ruling out the possibility of solid ovarian tumors or hematoma caused by ectopic pregnancy, which may present similar ultrasonographic findings, is crucial. MOE may be misdiagnosed as a solid ovarian tumor due to its uniform internal structure on ultrasonography [2]. Even though there are only a few reported cases of rapidly growing tumors during pregnancy [12,13], acquiring information on the time course of ovarian enlargement may help with the differential diagnosis. In our case, there was no abnormal ovarian enlargement during infertility treatment at the previous clinic, and this information helped us to conclude that the possibility of a solid ovarian tumor was low. In this case, ectopic pregnancy was initially suspected since ovary enlargement was observed after ovulation. The diagnosis was subsequently corrected, as ovarian hematomas caused by ectopic pregnancy often exhibit bizarre mixed echoes created by blood clots in a reticular pattern [14]; in contrast, MOE depicts a relatively uniform structure with the follicles displaced in the periphery of the ovary. Moreover, hematoma is likely to involve pressure pain, whereas MOE without torsion is asymptomatic. We were able to exclude ectopic pregnancy by observing the ultrasonographic characteristics, which confirmed that the GS was in the uterine cavity, and considering that the incidence of heterotopic pregnancy is relatively low [15]. In addition to ultrasonography, MRI as a combined diagnostic method is useful [10,16]. MRI of MOE shows unilateral ovarian enlargement with a teardrop configuration and T2 hyperintensity, which may provide additional clues for diagnosis [7,9,10,16]. The use of MRI in early pregnancy may be limited. However, MRI is valuable in cases where patients are symptomatic and decision-making for treatment is required or diagnostic uncertainty requires confirmation, as in the present case.

Laparoscopic surgery can be safely performed with less stress on the mother using minimally invasive procedures [4,17,18]. Treatment options include conservative management and surgery. However, considering fertility and its potential impact on pregnancy, especially during gestation, it is preferable to focus on ovarian preservation through close observation when patients are asymptomatic, or if symptomatic, detorsion and either wedge resection of the large part of the ovary or fixation should be considered [5,19]. In this case, we performed detorsion of the ovary coupled with a small biopsy incision to confirm the diagnosis and rule out malignancy, with the key goal of preserving the ovary and avoiding a second surgery. The biopsy was performed as rapid progress of both benign and malignant ovarian tumors has been reported previously [12,13]. As a result, the ovarian size reduced in a short time, which may have contributed to the prevention of torsion reoccurrence throughout the pregnancy. Regarding the speculation that the cause of MOE may be venous and lymphatic drainage obstruction and that the obstruction may worsen during pregnancy, drainage through the incision may have also helped.

Macroscopically, the ovarian surface in cases of MOE appears as a smooth, enlarged, white structure, whereas the cut surface is greyish-white and has a gelatinous consistency [1,5]. Pathologically, follicles are found in the peripheral cortex of the ovary, as seen on ultrasonography and MRI, and diffuse edema is observed in the stroma. Dilatation of the lymphatic vessels may also be noted. No inflammatory cell infiltration is observed in the edematous stroma [1,2,4]. In cases where the diagnosis is inconclusive, it may be essential to initially limit the intervention to biopsy alone and plan secondary treatment based on histopathological findings.

## Conclusions

Herein, we report a case of MOE involving ovarian torsion during pregnancy after ovarian stimulation with clomiphene citrate. Successful diagnosis was made in the early stage of the first trimester using imaging diagnostics, including ultrasonography and MRI. The patient underwent laparoscopic surgery as ovary conservative treatment, and the pregnancy outcome was uneventful.

Given the rarity of MOE during pregnancy, diagnosis can be challenging. However, by utilizing ultrasound and MRI findings, the pre-diagnostic probability can be enhanced. Preservation of the ovary is important in the treatment of MOE. Careful observation is recommended if the patient is asymptomatic. If symptomatic, performing detorsion with drainage of the edema via a small incision may be an effective method of size reduction in MOE and might even prevent torsion reoccurrence throughout the pregnancy. Preserving the ovary as much as possible is desirable in patients with MOE.
